# Building Polymeric Framework Layer for Stable Solid Electrolyte Interphase on Natural Graphite Anode

**DOI:** 10.3390/molecules27227827

**Published:** 2022-11-13

**Authors:** Yunhao Zhao, Yueyue Wang, Rui Liang, Guobin Zhu, Weixing Xiong, Honghe Zheng

**Affiliations:** 1 College of Energy, Soochow University, Suzhou 215006, China; 2 Sunwoda Electronic Co., Ltd., Shenzhen 518100, China; 3 Suzhou Huaying New Energy Materials and Technology Co., Ltd., Suzhou 215100, China

**Keywords:** natural graphite, artificial SEI, in situ polymerization, lithium-ion batteries

## Abstract

The overall electrochemical performance of natural graphite is intimately associated with the solid electrolyte interphase (SEI) layer developed on its surface. To suppress the interfacial electrolyte decomposition reactions and the high irreversible capacity loss relating to the SEI formation on a natural graphite (NG) surface, we propose a new design of the artificial SEI by the functional molecular cross-linking framework layer, which was synthesized by grafting acrylic acid (AA) and N,N′−methylenebisacrylamide (MBAA) via an in situ polymerization reaction. The functional polymeric framework constructs a robust covalent bonding onto the NG surface with —COOH and facilitates Li^+^ conduction owing to the effect of the —CONH group, contributing to forming an SEI layer of excellent stability, flexibility, and compactness. From all the benefits, the initial coulombic efficiency, rate performance, and cycling performance of the graphite anode are remarkably improved. In addition, the full cell using the LiNi_0.5_Co_0.2_Mn_0.3_O_2_ cathode against the modified NG anode exhibits much-prolonged cycle life with a capacity retention of 82.75% after 500 cycles, significantly higher than the cell using the pristine NG anode. The mechanisms relating to the artificial SEI growth on the graphite surface were analyzed. This strategy provides an efficient and feasible approach to the surface optimization for the NG anode in LIBs.

## 1. Introduction

In the past decades, graphite materials dominant for use as the anode in lithium-ion batteries (LIBs) [1,2,3]. Despite Si-based anodes, such as nano Si particles and SiO_x_, which attract a lot of attention in academic circles, graphite is still playing the most important role [4,5,6]. Artificial graphite (AG) is obtained from coke via ultra-high-temperature graphitization, with huge electricity consumption and severe environmental pollution [7,8]. By contrast, natural graphite (NG) is a natural resource with an ideal crystal structure. As a viable alternative to AG material, it contributes to significantly low costs and low carbon emissions [9,10]. However, some critical issues have long been the barriers hindering the wide use of NG materials, such as the large specific surfaces and high surface activity, much more internal voids, high expansion effects, etc. On the one hand, the SEI layer, resulting from the electrolyte decompositions at the electrode/electrolyte interface, consumes active Li-ions, resulting in a high irreversible capacity loss and low initial coulombic efficiency (ICE) in the first cycle [11]. On the other hand, the naturally grown SEI film, consisting mainly of inorganic lithium salts, such as LiF, Li_2_O, and Li_2_CO_3_, is quite brittle, and its damage and repair continuously rapture the active Li−ions, which eventually leads to a capacity failure of the cell [12,13]. For these reasons, natural graphite only accounts for about 20% of the anode material market in the LIB industry, and most of the adopted NG material is used by mixing with artificial graphite.

To solve all these issues, researchers have made many efforts and developed various interfacial decoration strategies. One is to use film-forming additives in the electrolyte, such as VC [14], FEC [15], VEC [16], LiBOB, and LiDFOB [17], etc. These additives can be reduced on the graphite surface prior to the electrolyte decomposition and, thus, promote the SEI stability [18,19,20]. Although effective, it is less feasible-facing with the NG anode. Moreover, significant side effects resulting from the residual additives retained in the electrolyte lead to the sustained growth of the SEI during the battery operation [10]. In the LIB industry, NG material is subjected to asphalt cladding and high-temperature carbonization [21]. It not only reduces the specific surface area of the graphite material, but it also improves the graphite conductivity [22]. However, serious environmental pollution and the accompanied high electricity consumption are the two biggest problems in its production. The pre-lithiation of the NG anode can compensate for the active Li-ion loss during the SEI formation [23,24]. However, it is hard to realize because lithium metal powder presents a challenge in the operation. The other approach is to construct the artificial SEI layer on the surface of the NG. Inorganic oxides, such as TiO_2_ [25], Al_2_O_3_ [26], TiNb_2_O_7_ [27], and Li_4_Ti_5_O_12_ [28], have been utilized as inert coatings on graphite surfaces to suppress the interfacial side reactions and enhance the SEI stability. However, the inorganic coatings are brittle and tend to crack and fall off from the graphite. Therefore, the long-term stability of the graphite material is always poor. By contrast, functional organic molecules have abundant groups that are easy to be grown on the graphite surface. More importantly, the protective functional layer acts as the SEI precursor, contributing to the formation of a stable, uniform, and Li-conductive SEI on the graphite. In addition, the specific surface and surface activity of NG can be simultaneously reduced by inserting organic molecules into the pores and voids. In recent years, various organic molecules have been attempted on Si and NG surfaces [10,29,30,31]. As expected, the high ICE of the electrode is obtained with a decrease of the active Li-ion loss in the cell formation.

**Figure 1:**
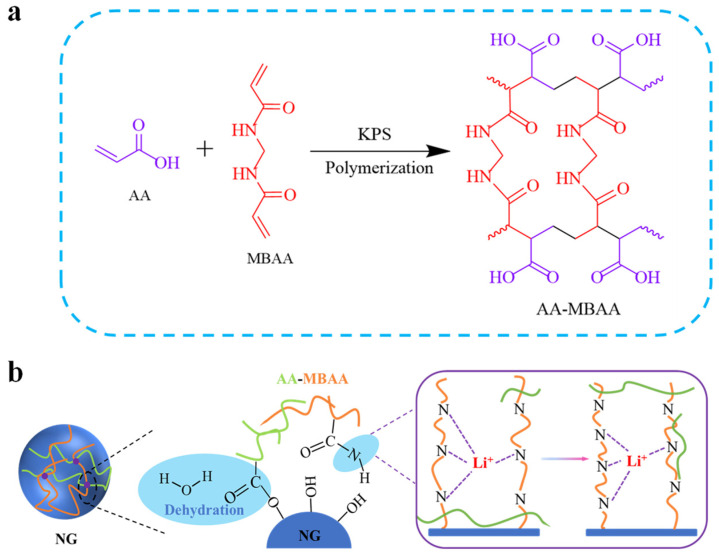
(**a**) Schematic for the AA–MBAA cross-linking framework by in situ polymerization of the AA and MBAA, and (**b**) a diagram of the function mechanism of the AA–MBAA polymer on the NG surface.

In this work, we proposed a new design of the artificial interface on the NG surface. Specifically, the cross-linking functional polymeric layer was copolymerized in water with N,N′-methylenebisacrylamide (MBAA) and acrylic acid (AA) via a free-radical initiated polymerization. Potassium persulfate (KPS) was adopted as the initiator for the in situ polymerization on the NG surface (Figure 1a). The AA molecule is widely used as an organic monomer, for it contains an abundant C=C bond and —COOH group with the role to establish covalent bonding with the NG surface, while MBAA is an excellent cross-linking agent for forming a cross-linked framework for conductive hydrogels [32], biocatalysts, and biosensors [33,34]. Furthermore, the free radicals were generated through the oxidation and reduction effect of the initiator, KPS, at 80 °C, thereby opening the double bonds in the monomer molecules and carrying out additional polymerization reactions between the molecules to form a macromolecular polymer. The copolymerization between the MBAA and AA forms a polymeric framework containing abundant functional groups (—COOH and —CONH). The —COOH groups can establish robust covalent bonding with the —OH groups on the NG surface by dehydration reactions, while the —CONH groups help to facilitate ionic diffusion by jumping between the N atoms, as illustrated in Figure 1b. Therefore, the cross-linking polymer layer acts as the SEI precursor on the NG surface and contributes to a stable, uniform, and ion-conductive SEI. As a consequence, at the optimized AA–MBAA (2 wt%) content on the NG surface (marked as NG@AM−2%), the overall electrochemical performance of the NG anode was remarkably improved. The ICE, rate capability, and cycling stability of the NG were greatly enhanced. The full cell with the NCM523 cathode also exhibited improved reversible capacity and long lifespan. The results demonstrate that the in-situ polymerization of functional molecules on the electrode surface is a promising strategy for developing high-performance NG materials used in LIBs.

## 2. Results and Discussion

### 2.1. NG@AA−MBAA (NG@AM) Electrodes Materials Characterization

**Figure 2:**
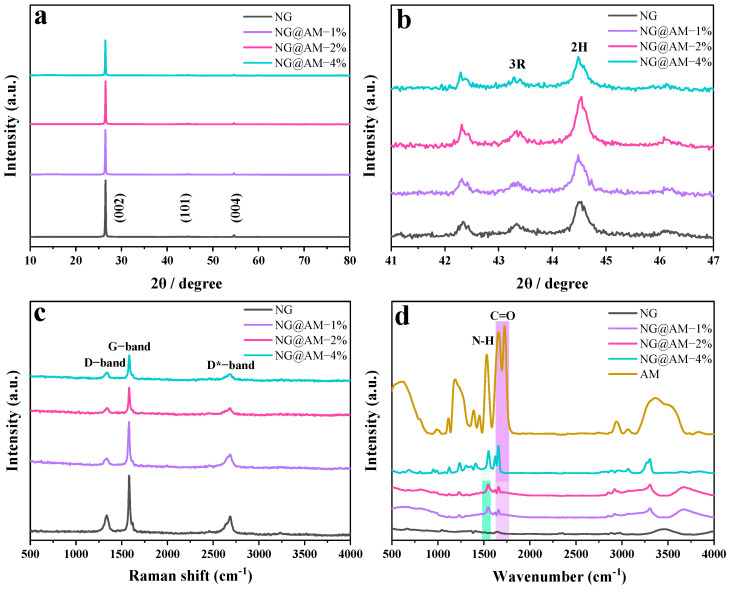
(**a**) XRD patterns and (**b**) enlarged image of XRD patterns of the (101) diffraction peak. (**c**) Raman spectra and (**d**) FTIR spectra of the NG grafted with different amounts of the AM polymer.

The crystalline structures of the NG implanted with different contents of the AM framework are compared with X-ray diffraction (XRD) in Figure 2a. The typical diffraction peaks are identical due to the low content of the AM polymeric layer. The only difference lies in the intensity of the characteristic (002) peak, which is gradually reduced with the increasing interfacial AM. The obvious decrease in the peak intensity reveals that the AM polymer layer is applied on the NG surface. Meanwhile, the diffraction peak (2H), relating to the hexagonal graphite (101) at 44.5° and the 3R peak corresponding to the rhombohedral graphite (101) at 43.5°, is clearly seen in Figure 2b, showing the adopted NG has hexagonal and rhombohedral crystal structures [35,36].

Figure 2c displays the Raman spectra of the NG samples implanted with different contents of the AM framework. Three characteristic peaks at 1340, 1580, and 2685 cm^−1^ are observed for the pristine NG, corresponding to the D−band, G−band, and D*−band, respectively [37]. The D−band at 1340 cm^−1^ is related to the disordered vibration of carbon, while the G−band at 1580 cm^−1^ is attributed to the tension vibration from sp^2^ hybrid carbon atoms [9]. The intensity ratio of the I_D_/I_G_ is related to the graphitization degree of the graphite. Interestingly, the I_D_/I_G_ ratio for the AM-decorated NG (0.466~0.567) is higher than that of the pristine NG (0.415). It indicates that the interfacial AM layer changes the orientation of the carbon atoms on the graphite surface. The decrease of all the characteristic peaks with the increasing AM content reveals the homogeneous distribution of the AM layer on the graphite surface.

The FTIR spectra were also analyzed to further verify the AM layer on the NG surface. For the pure MBAA substance (Figure S1), the typical peaks at 3308 cm^−1^ (the secondary amide N–H stretching vibration), 1659 cm^−1^ (the secondary amide C=O stretching vibration), 1629 cm^−1^ (the C=C stretching vibration), and 1546 cm^−1^ (the N–H bending vibration) were observed [34,38]. For the synthesized AM polymer, the characteristic peaks were attributed to the N–H bond and C=O bond. In addition, the peak at 1723 cm^−1^, corresponding to the C=O bond, was derived from the —COOH of the AA molecules. The disappearance of the C=C bond at 1629 cm^−1^ confirmed the successful polymerization between the AA and MBAA molecules [32]. For the NG samples grafted with the AM polymeric framework, most of the characteristic AM peaks were detected (Figure 2d), and the intensity grew with the increasing content of the interfacial AM polymer. Again, it demonstrated that the synthesized AM polymer was uniformly developed on the NG surface. XPS spectra of the pristine NG and the NG@AM−2% samples were analyzed to compare their interfacial differences. The C 1 s spectra obtained from the pristine NG (Figure S2a) showed several peaks at 284.8, 285.6, 287.1, and 289.1 eV, corresponding to the C–C, C–O/C–OH, C=O, and CO_3_^2−^ species, respectively [29,39]. As for the NG@AM−2% sample (Figure S2b), a new peak appeared at 286.5 eV, corresponding to the C–N bonds from the —CONH groups [40]. Meanwhile, the appearance of the C–N bond at 401 eV was observed from the N 1 s spectra of the NG@AM−2% sample (Figure S2d). The appearance of the C–N bond manifested that the AM polymer was successfully grown on the NG surface.

**Figure 3:**
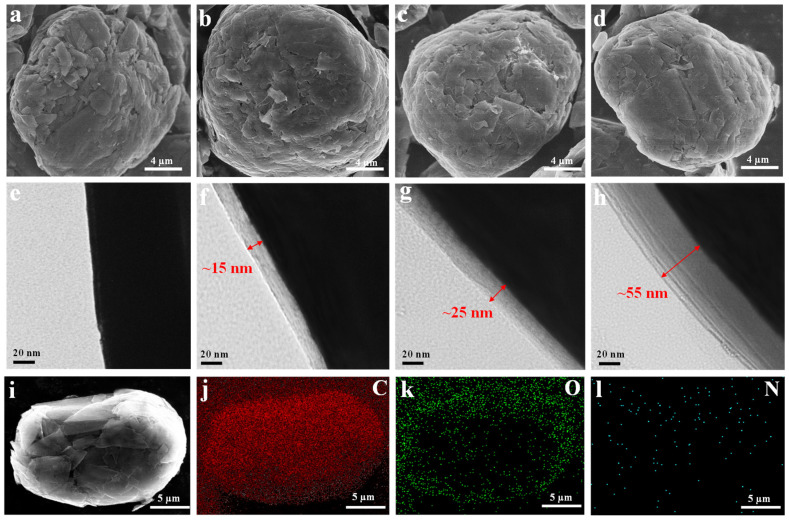
The SEM and TEM images of the pristine NG (**a**,**e**), NG@AM−1% (**b**,**f**), NG@AM−2% (**c**,**g**), and NG@AM−4% (**d**,**h**), respectively. The EDS mappings of electrode powder, the NG@AM−2% (**i**), C−mapping (**j**), O−mapping (**k**), and N−mapping (**l**).

The SEM and TEM images of the NG with different contents of the AM layer are depicted in Figure 3. From the SEM images, the dimension and morphology of the graphite particles are not considerably changed by the implanted AM polymer layer. The difference lies in the edges of the pristine NG being distinct and sharp, whereas the edges of the NG grafted by the AM polymer layer appear to be vague and smooth (Figure 3a–d). From the corresponding TEM images (Figure 3e–h), the synthesized AM layer is seen uniformly grown on the NG surface. The thickness varies from 15 to 55 nm, according to the content of the applied AM layer. Figure 3i–l shows the elemental mapping of the NG@AM−2% sample. It is seen that the NG particle is oval-shaped with uniformly distributed C, O, and N elements, but the content of N is a little less due to the very low MBAA content, implying the uniform distribution of the functionalized AM framework on the NG surface.

### 2.2. Electrochemical Properties of NG@AM Electrodes

**Figure 4:**
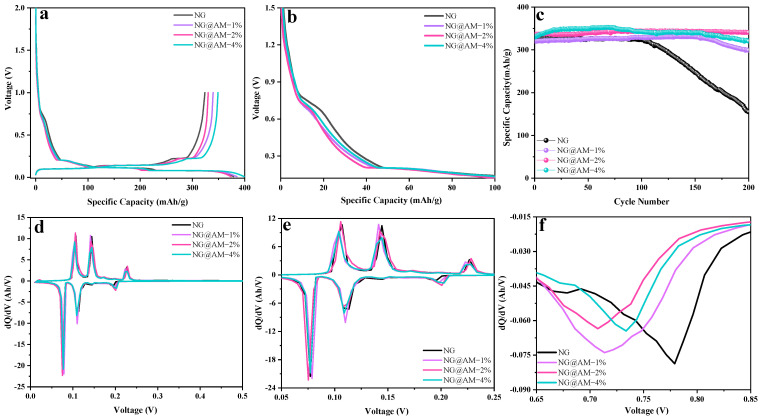
(**a**) The first charge/discharge curves and (**b**) the corresponding enlarged detail image of the charge curves for different NG electrodes. (**c**) The long-term cycling behavior for different NG electrodes. (**d**) The first differential capacity plots (dQ/dV versus the electrode potential) for different NG electrodes and the corresponding enlarged view of (**e**) oxidation/reduction peaks and (**f**) details around 0.78 V of differential capacity plots.

The electrochemical properties of the NG anodes with different contents of the AM layer are compared in Figure 4. From the first charge/discharge curves of the graphite anodes at 0.05 C in Figure 4a, all the curves exhibit similar voltage platforms corresponding to the Li-ion intercalation/deintercalation into/from the graphite anodes. The irreversible platform for the pristine NG at ~0.7 V is associated with the electrolyte decomposition and SEI formation. The applied AM layer is able to reduce the irreversible plateau by suppressing the interfacial irreversible reactions (Figure 4b). Table S1 shows the charge/discharge capacity and corresponding ICE for the NG anodes with different contents of the AM layer. Compared to the pristine NG anode, the interfacial AM layer contributes to a remarkable enhancement of the ICE and reversible capacity. For the pristine NG anode, the charge and discharge capacities are 378.52 and 323.82 mAh g^−1^, corresponding to the ICE of 85.55%. For the NG@AM−2% anode, the charge and discharge capacities of 398.92 and 356.28 mAh g^−1^ were obtained, corresponding to the ICE of 89.31%. Apparently, the cross-linking AM layer effectively inhibited the side reactions caused by the electrolyte component decomposition on the NG surface, contributing to the significant enhancement of the ICE. Considering that the irreversible capacity of the NG anode is closely related to its specific surface area, we determined the specific surface area from the nitrogen adsorption/desorption isotherms, as shown in Figure S3. For the pristine NG, NG@AM−1%, NG@AM−2%, and NG@AM−4%, the BET surface area was obtained to be 6.90, 4.06, 3.74, and 4.20 m^2^ g^−1^, respectively. Obviously, the specific surface area of the NG anode can be significantly reduced by the AM filling and coverage. Figure 4d displays the differential capacity plots (dQ/dV versus the electrode potential) in the first cycle. As shown in Figure 4e, three characteristic cathodic reduction peaks at 0.20 V, 0.11 V, and 0.08 V are associated with the formation of 4−stage, 2−stage, and 1−stage graphite intercalation compounds (GICs), respectively [41]. Meanwhile, the corresponding three anodic oxidation peaks are related to the Li-ion deintercalation from the graphite anodes. Figure 4c depicts the long-term cycling performance of the NG anode with different contents of the AM layer at 25 °C. It is seen that the pristine NG anode underwent a capacity decline after 100 cycles. By contrast, after applying the AM layer, a much-improved capacity retention of the electrode was observed after 200 cycles. The NG@AM−2% anode retained a reversible capacity of ~340 mAh g^−1^ after 200 cycles. Almost no capacity loss was seen, showing the excellent cycling stability of the NG electrode. As shown in Figure 4f, the irreversible cathodic peak at ~0.78 V originated from the electrolyte decomposition and SEI formation on the NG surface. The applied AM layer reduced the intensity of the 0.78 V peak while moving the peak to a lower potential, revealing the interfacial activity of the NG anode as decreased by the AM polymer coverage.

### 2.3. Kinetics Analysis

**Figure 5:**
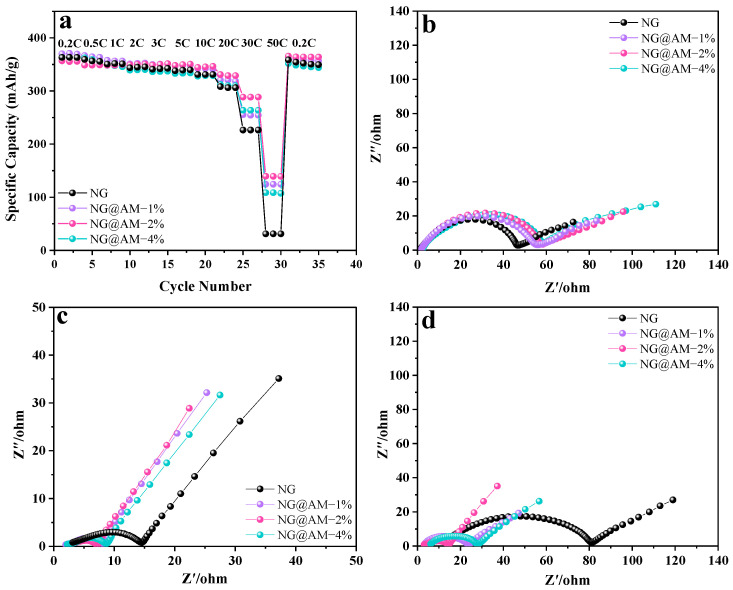
(**a**) Rate capability of the NG anodes with different contents of the AM layer. Nyquist plots of the different NG anodes at various electrochemical stages: (**b**) after cell formation, (**c**) after the rate test, and (**d**) after 200 cycles.

To investigate the kinetics of the NG anodes with different contents of the AM layer, the rate capability and the EIS spectra at different electrochemical stages were collected, as shown in Figure 5. The rate capability of the NG anodes with different contents of the AM layer is compared in Figure 5a. The NG@AM−2% anode was able to deliver a specific capacity of ~140 mAh g^−1^ at 50 C, significantly higher than the pristine NG anode. It shows that the applied AM polymer layer improved the electrochemical kinetics of the NG anode.

The equivalent circuit (Figure S4) is composed of the Ohmic resistance (R_S_), the SEI impedance (R_SEI_) in the high-frequency region, the charge transfer resistance (R_ct_) in the medium-frequency region, and the Warburg impedance in the low-frequency region. In all the Nyquist plots, the two semicircles with a sloped line from the high- to the low-frequency region correspond to the SEI resistance (R_SEI_), the charge transfer resistance (R_ct_) at the graphite/electrolyte interface, and the Li^+^ diffusion within the electrode (Z_W_), respectively [42]. From Figure 5b and Table S2, we see a slight increase in the NG impedance, with increasing contents of the AM layer after the cell formation. However, after the rate test (Figure 5c and Table S3), the R_SEI_ and R_ct_ of the NG anodes grafted with the AM polymer layer were significantly decreased. It well-explains the improved rate capability of the AM decorated samples. After 200 cycles, the R_SEI_ and R_ct_ for the pristine NG anode exhibited remarkable growth, while the AM-decorated NG anodes showed an impedance decrease (Figure 5d and Table S4). For the pristine NG, the continuous growth and thickening of the SEI layer are known to be responsible for the impedance rise with the electrochemical cycling. For the AM-decorated NG anodes, AM polymer participates in the SEI formation and facilitates Li-ion transportation at the electrode/electrolyte interface. Therefore, the NG@AM−2% anode exhibited the lowest impedance after the rate and cycling test.

The diffusion coefficients of Li^+^ for the NG anodes with different contents of the AM layer were calculated by the following Equation [43]:

(1) $$D=\left(R^{2}T^{2}\right)/\left(2A^{2}N^{4}F^{4}C^{2}\sigma^{2}\right)$$

(2) Z′∝σω−12

where *D* is the diffusion coefficient of Li^+^, *R* is the gas constant, *A* represents the surface area of the anode, *N* means the electron number for every crystalline cell during Li^+^ insertion, *F* is the Faraday constant, *C* is the Li^+^ concentration, *σ* indicates the Warburg factor, *Z′* denotes the substantive part of the resistance in the low-frequency region, and *ω* is the corresponding frequency. The Li^+^ diffusion coefficients after the rate test for the pristine NG, NG@AM−1%, NG@AM−2%, and NG@AM−4% were calculated to be 2.05 × 10^−12^, 6.33 × 10^−12^, 7.12 × 10^−12^, and 5.21 × 10^−12^ cm^2^ s^−1^, respectively. After 200 cycles, the Li^+^ diffusion coefficients were 6.75 × 10^−13^, 3.62 × 10^−12^, 5.04 × 10^−12^, and 3.57 × 10^−12^ cm^2^ s^−1^, respectively. Obviously, the D_Li_^+^ of the NG@AM−2% is higher than the other samples. The enhanced D_Li_^+^ by the AM wrapping on the NG surface is attributed to the following reasons: (1) The AM polymer layer is in situ synthesized on the NG surface, which could effectively inhibit the electrolyte decomposition and the thickening of the SEI layer. (2) The —CONH group from the MBAA facilitates Li transportation. (3) The affinity between the functional AM layer and the graphite surface is conducive to reducing the Li-ion transport barrier at the electrode/electrolyte interface.

### 2.4. Interface Evolution Analysis of Cycled Electrodes

**Figure 6:**
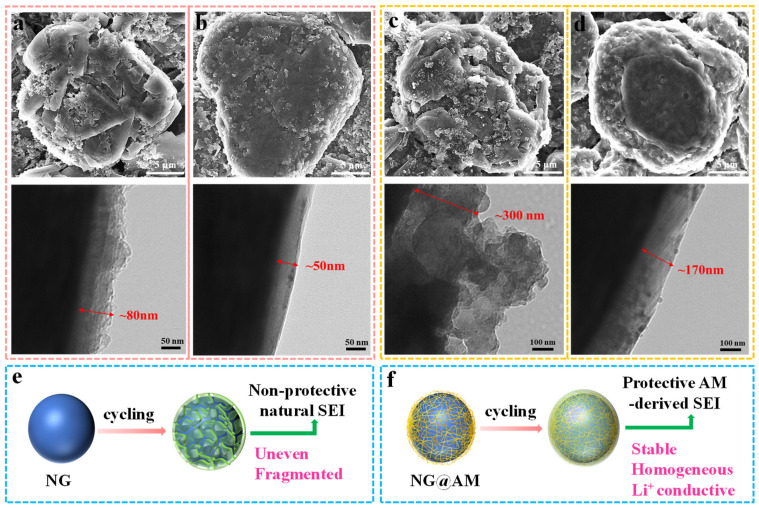
The SEM and TEM images of the (**a**,**c**) pristine NG anode and (**b**,**d**) NG@AM-2% anode at different electrochemical stages: (**a**,**b**) after formation and (**c**,**d**) after 200 cycles. Schematics of the SEI evolution with electrochemical cycles: (**e**) the pristine NG and (**f**) NG@AM-2% anodes.

The morphological evolution of the pristine NG and NG@AM−2% anodes was investigated at different electrochemical stages by SEM and TEM observations. After three formation cycles, a loosely, unevenly distributed and thick SEI layer (~80 nm) on the pristine NG is observed (Figure 6a). In contrast, a thin (~50 nm), uniform passivation layer on the NG@AM−2% surface is seen, as shown in Figure 6b, manifesting the effective protection of the applied AM layer. After 200 cycles, the pristine NG electrode was fully wrapped with a thick, bumpy, and fragmented surface layer derived from the severe by-product accumulation. The thickness of the surface layer even gets to ~300 nm (Figure 6c), indicating the sustained side reactions at the electrode/electrolyte interface. Due to the volume change of the graphite particle during the Li intercalation/deintercalation, the naturally grown SEI aroused from the electrolyte decomposition is prone to crack (Figure 6e). As a consequence, continuous interfacial decompositions of the electrolyte contribute to the consecutive growth of the SEI layer. By contrast, on the NG@AM−2% surface, only ~170 nm of the SEI layer is observed (Figure 6d). The surface film is not only thinner but also very uniform and compact. In this sense, the AM−2% polymer layer effectively protected the graphite by constructing a homogeneous, stable SEI layer, as shown in Figure 6f. Figure S5 shows the FTIR spectra of the NG anodes without and with the 2% AM surface layer at different electrochemical states. The typical peaks at 694, 872, 1444, and 1507 cm^−1^ were assigned to the Li_2_CO_3_, and the bands at 1063 cm^−1^ (C–O) and at 1640 cm^−1^ (C=O) were ascribed to the lithium alkyl carbonate (ROCO_2_Li), and the peak at 1288 cm^−1^ (P=O) corresponded to the decomposition products of the lithium hexafluorophosphate (LiPF_6_) [44,45]. For the pristine NG anode, the intensity of the P=O bond and Li_2_CO_3_ significantly increases after 200 cycles. However, the intensity growth of the P=O bond and Li_2_CO_3_ peaks for the NG@AM−2% anode after 200 cycles is less obvious. It further confirms that the artificial SEI converted from the cross-linking AM layer suppressed the electrolyte decompositions in the electrochemical cycles.

**Figure 7:**
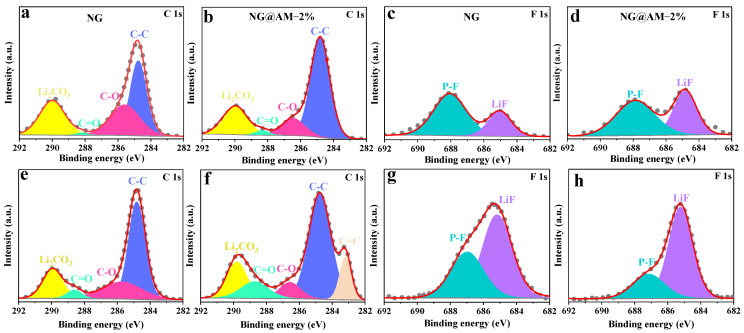
The fitted XPS spectra for the pristine NG and NG@AM-2% electrodes (**a**–**d**) after formation and (**e**–**h**) after 200 cycles: (**a**,**b**,**e**,**f**) C 1 s and (**c**,**d**,**g**,**h**) F 1 s.

Figure 7 shows the fitted XPS spectra for the pristine NG and NG@AM−2% electrodes after cell formation and 200 cycles. For the C 1 s spectra, the peaks at 284.8, 286.5, 288.6, and 290 eV correspond to the C–C, C–O, C=O, and Li_2_CO_3_, respectively. For the pristine NG electrode, the intensity of the C=O and Li_2_CO_3_ increases with the electrochemical cycling, implying a continuous SEI growth due to the continuous interfacial electrolyte decompositions (Figure 7a,e). As for the NG@AM−2% electrode, a new peak at 283.1 eV is attributed to unsaturated C=C bonds. The intensity growth of the C=O and the C–O peak decrease are ascribed to the rich —COOH and —CONH inherited from the AM layer and reduced by the ROCO_2_Li product (Figure 7b,f). For the F 1 s spectra, the P–F and LiF are identified at 687.5 and 685.4 eV and are ascribed as the reduced product from the LiPF_6_ [46,47]. With the electrochemical cycling, it is seen that more LiF and less P–F is generated on the NG@AM−2% surface compared with the pristine NG anode after 200 cycles (Figure 7c,g). It indicates that the reduction reaction of the LiPF_6_ is suppressed by the AM−2% polymer layer (Figure 7d,h). In addition, the LiF is considered to be an excellent component for stabilizing the SEI layer [30,48]. Hence, the cross-linked AM layer plays a role in stabilizing the SEI layer by inhibiting the electrolyte decomposition reactions.

### 2.5. Full Cell Performance of NCM523//NG@AM

**Figure 8:**
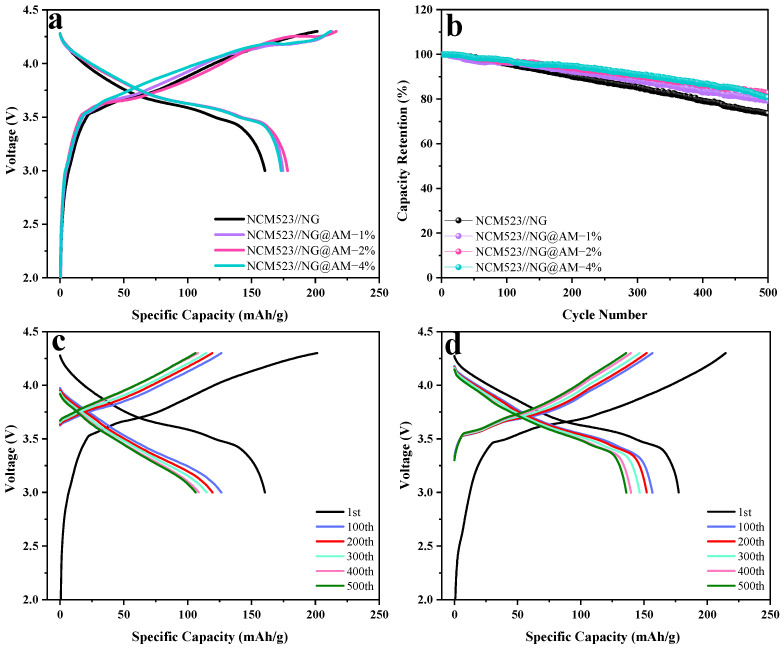
(**a**) The initial charge/discharge profiles of the full cells based on the NCM523 cathode and different NG anodes and (**b**) the cycling behavior. The discharge curves of the full cells with (**c**) the pristine NG anodes and (**d**) the NG@AM-2% anodes at different cycles.

To further validate the feasibility of the cross-linked AM layer on the graphite surface, full cells with NCM523 as the cathode were assembled. Figure 8a depicts the charge/discharge curves of the full cells using the NG anode with different contents of the AM layer. As shown in Table S5, the initial discharge capacity of the full cell using the pristine NG anode is 160.4 mAh g^−1^, corresponding to the ICE of 79.6%. The full cell using the NG@AM−2% anode demonstrates a reversible capacity of 178.0 mAh g^−1^ and an ICE of 82.35%. The increased capacity and ICE are due to the reduction of the irreversible capacity loss on the graphite anode by the protection of the AM layer. In Figure 8b, the long cycling performance of these full cells is compared. After 500 long cycles, a capacity retention of 82.75% was maintained for the full cells using the NG@AM−2% anode, which is significantly higher than that of 73.50% using the pristine NG anode. Of course, the enhanced cycling stability resulted from the stable SEI layer constructed by the cross-linked AM layer. As a consequence, the active Li^+^ consumption is suppressed, and the cycle life of the cell is prolonged. In addition, from the discharge curves of the full cells with the pristine NG and NG@AM−2% anodes at different cycles (Figure 8c,d), the IR drop was clearly suppressed with the applied AM layer. This well agrees with the improved electrochemical kinetics of the NG anode with the cross-linked AM layer.

Self-discharge is the phenomenon of the spontaneous depletion of the battery power [49]. To further compare the self-discharge performance of the full cells using the NG anode with different contents of the AM layer, voltage drops at different periods of time were measured. Figure S6 exhibits the voltage variations with the rest time for the full cells stored at 25 °C. The voltage drop of the full cell using the pristine NG anode is larger than the full cells using the AM-decorated graphite anodes. The K-value is calculated according to the voltage drop of the cell per time, and the results are compared in Table S6. For the full cell, 1.27 mV/h is obtained using the pristine NG anode. The K-value is significantly reduced for the full cells using the AM-decorated graphite anode. Moreover, the self-discharge rate (η) [50] is calculated according to the capacity retention during 8 days’ rest, as seen in Table S6. Similarly, η of the full cells using the AM-decorated NG anodes is significantly lower than that using the pristine NG anode at different conditions (25 °C and 50 °C). The significantly reduced self-discharge is related to the compact and stable artificial SEI layer developed with the help of the cross-linked functional AM surface layer. The stable and compact SEI layer is able to effectively prevent electron leakage from the graphite, thereby improving the self-discharge performance of the full cells.

## 3. Materials and Methods

### 3.1. Materials and Sample Preparation

Natural graphite (NG, 15–20 µm in diameter, and d_002_ = 0.3356 nm, with a specific surface area = 6.9 m^2^ g^−1^) was obtained from Suzhou Huaying New Energy Materials Co., Ltd., Suzhou, China. Acetylene black (AB, Denka Singapore Private Co., Ltd., Fukuoka, Japan) with an average particle size of 40 nm and a polyvinylidene fluoride (PVDF) binder (MW of ~600,000, Kureha Battery Materials Shanghai, Inc. Shanghai, China) that was purchased and used as the conductive additive and electrode binder, respectively. Acrylic acid (AA) monomer (99%) was purchased from Aladdin Bio-Chem Technology Co., Ltd., Shanghai, China. Potassium persulfate (KPS, >99%) and N,N′-methylenebisacrylamide (MBAA, >99%) were obtained from Sigma. All the materials were used as received without further treatment.

The 0.2 g MBAA was dissolved in 19.8 g of deionized to obtain a 1 wt% MBAA solution. A 4 g AA solution (5 wt%) was added into the 20 g 1 wt% MBAA solution (the weight ratio between the MBAA to the AA was 1:1). The obtained solution was neutralized (pH = 7) by adding an appropriate amount of Na_2_CO_3_ solution. Thereafter, 20 g of NG powder was dispersed into the solution under high-speed stirring. Then, 4 g of the KPS initiator solution (1 wt%) was introduced at 80 °C. The in situ polymerization between the AA and MBAA lasted for 2 h under 80 °C in an open container until the water solvent was completely evaporated with high-speed stirring. Finally, the obtained NG powder was treated under vacuum at 110 °C for 12 h in order to build a chemical bond between the AA–MBAA polymer and the graphite surface. The obtained NG samples were denoted as NG@AM−2%. In the same manner, the NG@AM−1% and NG@AM−4% samples were prepared.

### 3.2. Electrode Preparation and Electrochemical Testing

Electrochemical tests were carried out in CR2032-type coin cells assembled in a glove box (<0.5 ppm of oxygen and water, OMNI-LAB). For the half-cell assembly, the NG anode served as the working electrode and the Li foil as the counter electrode. Celgard 2500 was adopted as the separator between the two electrodes. The electrolyte was obtained from Capchem, China, consisting of 1 M of LiPF_6_ in a mixture of ethylene carbonate (EC)/ethyl methyl carbonate (EMC)/dimethyl carbonate (DMC) (1:1:1 in volume). Galvanostatic discharge/charge cycling of the cells between 0.01 and 1 V vs. Li/Li^+^ was tested on a Neware battery cycler (CT-4008) at room temperature. The rate capability was compared at 0.2 C charge (lithiation) and the various discharge (delithiation) rates from 0.2 C to 50 C, respectively. The long-term cycling performance of the NG anode was evaluated at a 0.2 C charge and 0.5 C discharge after the cell formation. Electrochemical impedance spectroscopy (EIS) was carried out on an electrochemical workstation (Zahner Elektrik IM6) over a frequency range between 0.01 Hz and 100 kHz with a voltage amplitude of 5 mV. Before the EIS measurement, the cells were controlled at a 60% depth of discharge (DOD) at 25 °C for at least 6 h.

The full cells were assembled with the NG anode and the NCM523 cathode. The cycling stability of the full cells was compared at a 0.5 C charge and 1 C discharge after the cell formation between 3 and 4.3 V at 25 °C. A self-discharge test of the full cell was determined by charging these cells to 4.3 V at a 0.2 C rate and resting for 8 days. The voltage drop was recorded at 24 h intervals.

### 3.3. Physical Characterizations

Scanning electron microscopy (SEM) and transmission electron microscopy (TEM) (FEI Tecnai HT7700 S-TWIN, 200 kV) were used to observe the morphology and microstructure of the graphite electrodes. The electrode powder samples for the TEM were prepared by ultrasonic dispersion in anhydrous ethanol and then dried onto a carbon film. Crystalline structures of the NG anodes were analyzed by X-ray diffraction (XRD) (Rint2000, Rigaku with Cu Kα), and the diffraction angle (2θ) was scanned between 10 and 80° with an increment of 3°/min. Raman spectra (Jobin YvonLab HR 800) were obtained with the 632.8 nm excitation laser. A Fourier transform infrared (FTIR) spectrometer (Bruker Optics, Tensor 27, Borken, Germany) was used to characterize the functional groups on the NG surface. X-ray photoelectron spectroscopy (XPS, Escalab 250Xi, Thermo Fisher, Waltham, MA, USA) was used to probe the chemical compositions on the NG surface at different electrochemical stages. The BET surface area was measured by nitrogen adsorption/desorption isotherms using an automatic adsorption system (BET, ASAP2460, Norcross, GA, USA). After electrochemical cycling, the electrodes retrieved from the cells were rinsed three times with a DMC solvent, and then we evaporated the solvent in a vacuum chamber for 2 h.

## 4. Conclusions

In summary, we have reported a cross-linked AM framework layer with an in situ polymerization reaction via free radical initiation. The artificial layer was grafted onto the NG surface with covalent bonding by dehydration reactions between —COOH groups of the AA and —OH groups. With this design, the instability of the SEI film on the graphite surface can be effectively solved. In addition, the —CONH group from the MBAA facilitated the Li^+^ transportation by jumping between the N atoms. The AM framework layer not only reduced the potential of electrolyte decomposition and SEI formation but also offset the internal stress with the good flexibility of the AM layer. Benefiting from all these merits, the NG anode with the artificial SEI layer exhibited a much-improved ICE, rate capability, and cycling stability. After the optimization, the NG@AM−2% demonstrated the best electrochemical properties in the half cell against the lithium. Furthermore, the full cells using the NCM523 cathode showed a similar improvement, demonstrating the practical use of the designed AM surface layer on the NG graphite. The full cell with the optimized NG@AM−2% anode not only achieved an improved ICE and reversible capacity but also retained a high capacity after 500 cycles. Therefore, building a polymeric framework layer for the stable solid electrolyte interphase via in situ polymerization of functional molecules provides a solution to the instability of the SEI film on the natural graphite anode.

## Data Availability

The datasets used and/or analyzed in the present study are available from the corresponding author upon reasonable request.

## Supplementary Materials

The following supporting information can be downloaded at: https://www.mdpi.com/article/10.3390/molecules27227827/s1, Figure S1: The FTIR spectra of the pure MBAA particles and AA–MBAA polymer; Figure S2: XPS spectra with fitted results for the pristine NG and NG@AM−2% electrodes: (**a**,**b**) C 1 s and (**c**,**d**) N 1 s; Figure S3: (**a**) The nitrogen adsorption/desorption isotherms and (**b**) the BET surface area of the pristine NG, NG@AM−1%, NG@AM−2%, and NG@AM−4% electrodes; Figure S4: The equivalent circuit for fitting the EIS; Figure S5: The FTIR spectra of the pristine NG and NG@AM−2% anodes at different electrochemical stages; Figure S6: Plot of voltage varies with the rest time for full cells with different NG anodes stored at 25 °C; Table S1. The charge/discharge specific capacity (CSC/DSC) and initial coulombic efficiency of different NG anodes in the first cycle; Table S2. The corresponding resistance values of the pristine NG and NG grafted with different amounts of the AM polymer anodes after the three formation cycles; Table S3. The corresponding resistance values of the pristine NG and NG grafted with different amounts of the AM after the rate test; Table S4. The corresponding resistance values of the pristine NG and NG grafted with different amounts of the AM polymer anodes after 200 cycles; Table S5. The first charge/discharge specific capacity, initial coulombic efficiency, and capacity retention (CR) after 500 cycles of full cells with different NG anodes; Table S6. The K-values and self-discharge rates (η) of full cells with different NG anodes at 25/50 °C.

## Sample Availability

Samples of the compounds natural graphite are available from the authors.
