# Supplementary material for: Building Polymeric Framework Layer for Stable Solid Electrolyte Interphase on Natural Graphite Anode
Source: Molecules. 2022 Nov 13;27(22):7827. doi: 10.3390/molecules27227827 (PMC9692837; doi:10.3390/molecules27227827)
Supplement: Supplementary file 1 [file molecules-27-07827-s001.zip › molecules-1992559-supplementary.pdf]

Supplementary Materials

Building Polymeric Framework Layer for Stable Solid Electrolyte Interphase on Natural Graphite Anode

Yunhao Zhao [1], Yueyue Wang [1], Rui Liang [2,*], Guobin Zhu [1,3,*], Weixing Xiong [1,3] and Honghe Zheng [1,3,*]

[1] College of Energy, Soochow University, Suzhou 215006, China
[2] Sunwoda Electronic Co., Ltd., Shenzhen 518100, China
[3] Suzhou Huaying New Energy Materials and Technology Co., Ltd., Suzhou 215100, China
* Correspondence: liangrui@sunwoda.com (R.L.); gbzhu@suda.edu.cn (G.Z.); hhzheng@suda.edu.cn (H.Z.)

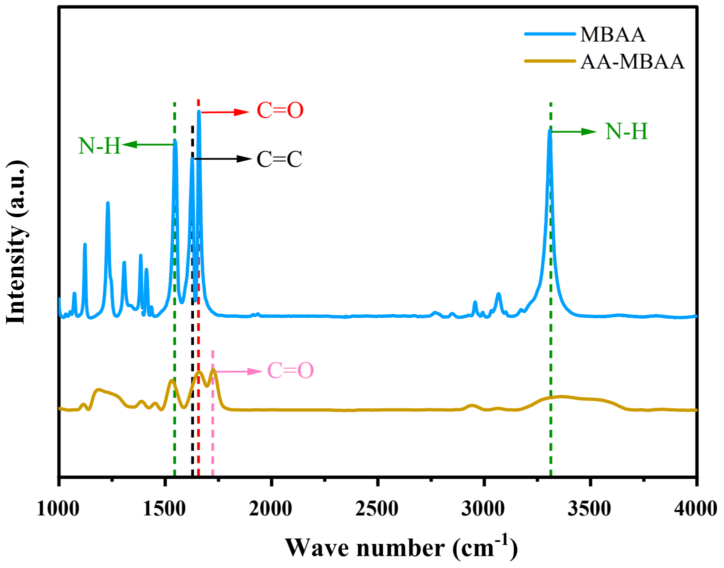

Figure S1. The FTIR spectra of the pure MBAA particles and AA-MBAA polymer.

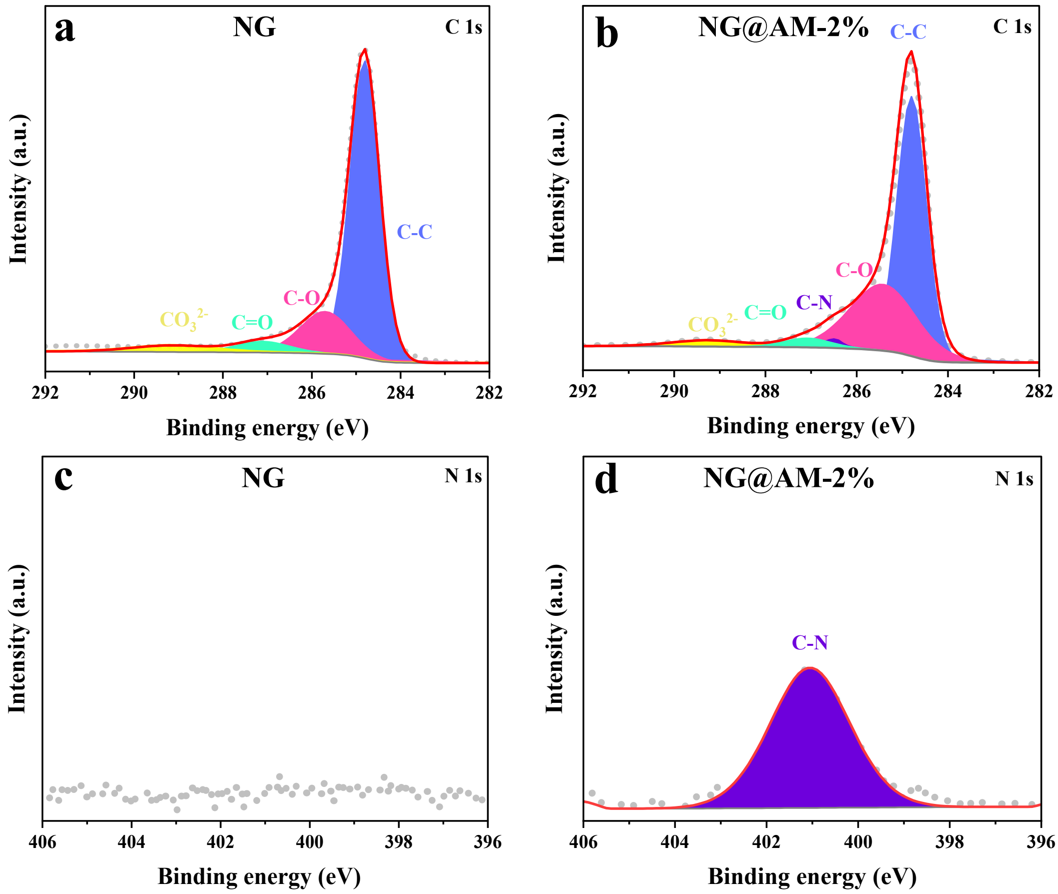

Figure S2. XPS spectra with fitted results for the pristine NG and NG@AM-2% electrodes: (a, b) C 1s and (c, d) N 1s.

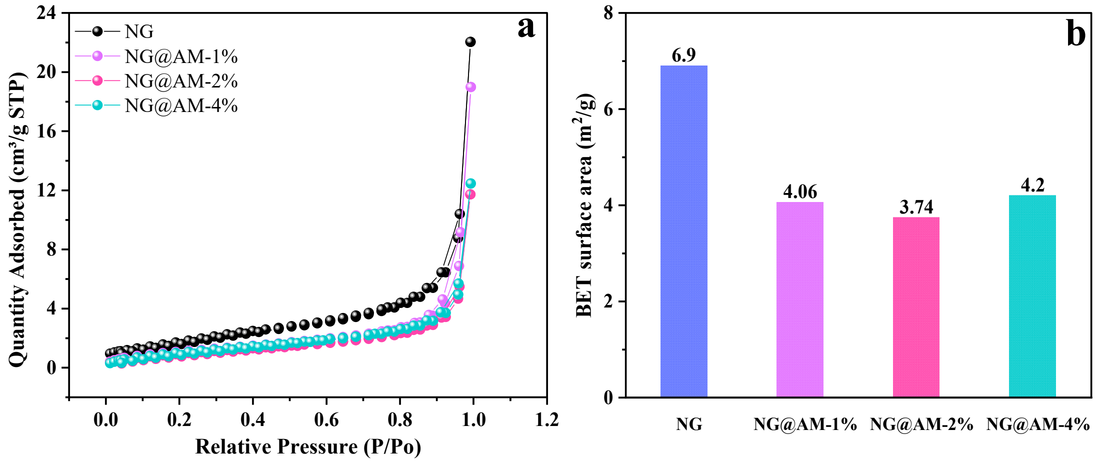

Figure S3. (a) The nitrogen adsorption-desorption isotherms and (b) the BET surface area of the pristine NG, NG@AM-1%, NG@AM-2% and NG@AM-4% electrodes.

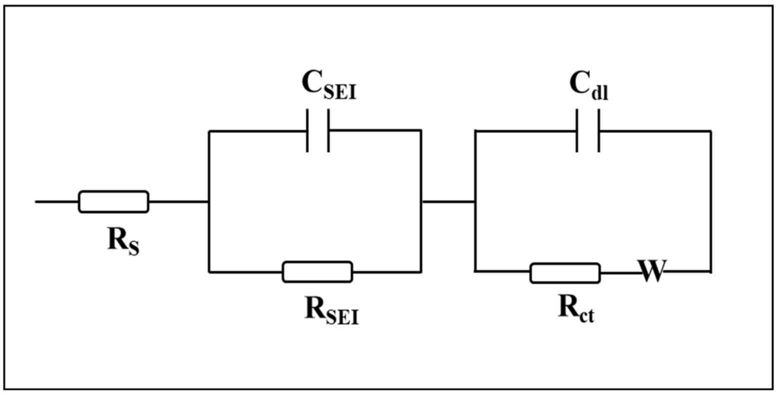

Figure S4. The equivalent circuit for fitting the EIS.

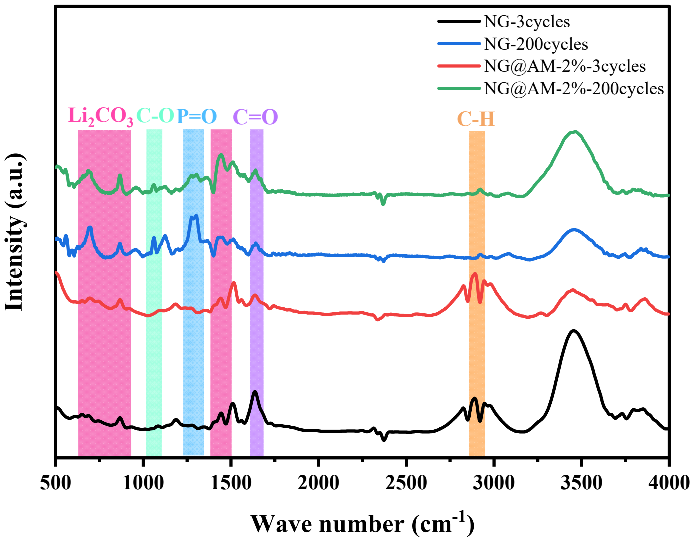

Figure S5. The FTIR spectra of the pristine NG and NG@AM-2% anodes at different electrochemical stages.

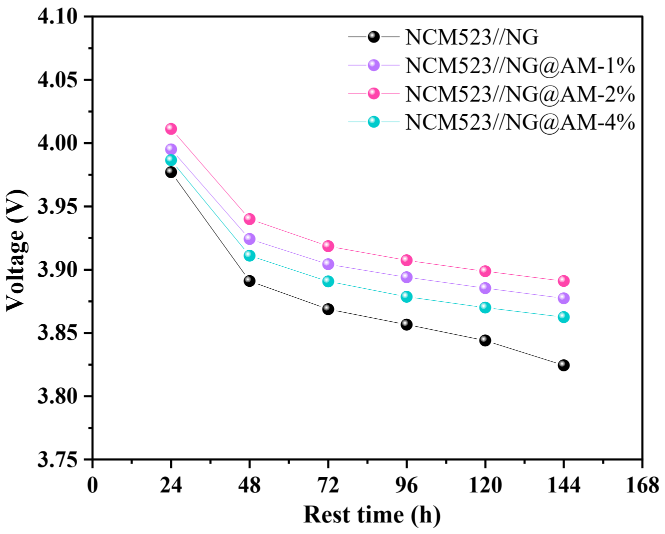

Figure S6. Plot of voltage varies with rest time for full cells with different NG anodes stored at 25°C.

Table S1. The charge/discharge specific capacity (CSC/DSC) and initial coulombic efficiency of different NG anodes in the first cycle.

Samples	CSC (mAh g^{-1})	DSC (mAh g^{-1})	ICE (%)
NG	378.52	323.82	85.55
NG@AM-1%	385.07	339.45	88.15
NG@AM-2%	398.92	356.28	89.31
NG@AM-4%	399.04	348.88	87.43

Table S2. The corresponding resistance values of the pristine NG and NG grafted with different amounts of the AM polymer anodes after the 3 formation cycles.

R (Ω cm^2)	R_S	R_{SEI}	R_{CT}
NG	1.24	4.13	39.48
NG@AM-1%	1.74	6.04	45.42
NG@AM-2%	1.48	9.73	42.64
NG@AM-4%	2.32	18.34	39.95

Table S3. The corresponding resistance values of the pristine NG and NG grafted with different amounts of the AM polymer anodes after the rate test.

R (Ω cm^2)	R_S	R_{SEI}	R_{CT}
NG	2.07	4.21	8.04
NG@AM-1%	1.59	2.16	4.30
NG@AM-2%	1.58	2.01	3.44
NG@AM-4%	1.61	2.87	4.87

Table S4. The corresponding resistance values of the pristine NG and NG grafted with different amounts of the AM polymer anodes after the 200 cycles.

R (Ω cm^2)	R_S	R_{SEI}	R_{CT}
NG	11.68	23.02	45.06
NG@AM-1%	2.79	8.95	12.18
NG@AM-2%	2.17	4.21	8.04
NG@AM-4%	5.41	6.41	15.48

Table S5. The first charge/discharge specific capacity, initial coulombic efficiency and capacity retention (CR) after 500 cycles of full cells with different NG anodes.

Samples	CSC (mAh g^{-1})	DSC (mAh g^{-1})	ICE (%)	CR (500th)
NG	201.5	160.4	79.60	73.50%
NG@AM-1%	212.9	174.6	82.01	79.23%
NG@AM-2%	216.4	178.2	82.35	82.75%
NG@AM-4%	211.9	173.2	81.74	80.73%

Table S6. The K-values and self-discharge rates (η) of full cells with different NG anodes at 25/50℃.

	NCM523//NG	NCM523//NG @AM-1%	NCM523//NG @AM-2%	NCM523//NG @AM-4%
K-25℃ (mV/h)	1.27	0.98	1.01	1.03
η-25℃ (%)	8.57	7.24	6.61	3.64
η-50℃ (%)	12.27	11.66	5.62	3.52
